# Crosstalk between PI3K/Akt and Wnt/β-catenin pathways promote colorectal cancer progression regardless of mutational status

**DOI:** 10.1080/15384047.2022.2108690

**Published:** 2022-08-09

**Authors:** Cassio Dejair Fleming-de-Moraes, Murilo Ramos Rocha, Josiane Weber Tessmann, Wallace Martins de Araujo, Jose Andres Morgado-Diaz

**Affiliations:** a Cellular and Molecular Oncobiology Program, Cellular Dynamic and Structure Group, Instituto Nacional de Cancer – INCA, Rio de Janeiro, Brazil; b Institute of Biological and Health Sciences, Federal Rural University of Rio de Janeiro, Rio de Janeiro, Brazil

**Keywords:** PI3K, Akt, Wnt, β-catenin, cell signaling, colorectal cancer

## Abstract

The PI3K/Akt and Wnt/β-catenin pathways play an important role in the acquisition of the malignant phenotype in cancer. However, there are few data regarding the role of the interplay between both pathways in colorectal cancer (CRC) progression. The mutational status and the clinicopathological characteristics of PI3K/Akt and Wnt/β-catenin pathways were accessed by bioinformatic analysis whereas that the impact of the interplay between the activity of both pathways to explain tumorigenic potential was performed in vitro using IGF-1 and Wnt3a treatments in CRC cell models. The mutational status of these pathways did not influence the survival of CRC patients, but an association between clinicopathological characteristics in patients with mutations in one, but not in both pathways was observed. A potentiating effect on the activation of both pathways and enhanced cellular migration and proliferation was observed when both pathways were activated simultaneously with IGF-1 and Wnt3a. In addition, these effects were hindered after pretreatment with LY294002, a specific PI3K inhibitor, suggesting some dependence between these two signaling cascades. Our findings show that, regardless of mutational status, there is an interplay between the activity of PI3K/Akt and Wnt/β-catenin pathways that contributes to events related to CRC progression and that the reversal of such events using a PI3K inhibitor highlights the value of targeting these pathways for potential directed therapies in CRC patients.

## Introduction

Colorectal cancer (CRC) is one of the leading causes of cancer-related deaths. Its high mortality rate is a consequence of various factors including the lack of apparent symptoms in early stages and deficits in cancer prevention strategies, particularly in developing countries.^1^ Indeed, the median 5-year survival rate is of approximately 66.4%, which is due to the majority of cases being diagnosed in a late incurable stage, when the effect of traditional cancer treatments is limited.^2^ Unveiling the mechanisms responsible for such limitations is crucial for identifying new predictive biomarkers of drug response that could improve the selection of patients sensitive to treatment.

The dysregulation of various signaling pathways plays a significant role in the development of CRC. Over activation of the Wnt/β-catenin signaling pathway is one of the most frequent alterations in CRC. Their abnormal activation caused by mutations in *APC, CTNNB1* or *AXIN2* reduces the capacity of the destruction complex, composed of APC, AXIN and GSK3β, to commit β-catenin to degradation. As a result, β-catenin accumulates in the nucleus, binds the TCF/LEF transcription factors and induces the expression of target genes that play key roles in tumor progression.^3^ Activation of this pathway contributes to the onset and progression of more than 90% of CRC cases.^4^ Studies show that 20% of CRC patients present mutations in genes of the PI3K pathway such as, *PIK3RI, PI3KCA* and *PTEN*.^5,6^ In particular, *PI3KCA* plays a role in the activation of PI3K/Akt signaling to promote proliferation, cell growth and survival. Drugs targeting PI3K/Akt activity are currently in clinical trials, however, CRC patients exhibit some resistance to these drugs.^7–10^ In addition, as inappropriate activation of the Wnt/β-catenin pathway was first linked to CRC, there is an intense interest in developing effective inhibitors.^11^ Unfortunately, the activation or inhibition of a single signaling pathway is unlikely to result in a substantial improvement in disease progression given the co-activation of numerous oncogenic pathways in this cancer. For instance, it was shown that mutations in genes frequently altered in CRC, including *KRAS, TP53*, or *PIK3CA*, do not predict the response to PI3K/Akt pathway inhibitors. Interestingly, multivariate analysis indicated that *APC* mutations are a risk factor for patients treated with these inhibitors, which suggests that the activation of the Wnt/β-catenin pathway could participate in the mechanism of resistance to PI3K/Akt inhibitors.^12^

The individual participation of the PI3K/Akt and Wnt/β-catenin pathways during the CRC progression is well established.^13,14^ However, recent studies have shown that an interaction between these two pathways contributes to the progression of various cancer types, including the ovary and pancreas.^15,16^ In CRC patient samples, the presence of nuclear β-catenin and an active PI3K/Akt pathway was associated with an increased risk of metastasis, suggesting a crosstalk between these pathways.^17^ However, little is known about how these pathways interact to contribute to CRC progression.

This study was designed to identify players that mediate the interaction between PI3K/Akt and Wnt/β-catenin pathways or mutational alterations that might be useful in predicting responses to treatment using modulators of these pathways and to determine their viability as therapeutic targets for CRC treatment.

## Results

### Mutational status of WNT and PI3K pathways in CRC patients

We analyzed the presence of mutations in the main components of the Wnt/β-catenin (*APC, CTNNB1, AXIN1* and *AXIN2*) and PI3K/Akt pathways (*PI3KCA, PTEN, PIK3R1* and *PIK3R2*) in several open-access CRC clinical cohorts. Of the 705 patients included in the study, 397 (56.3%) had mutations in the *APC* gene; 11 (1.6%) patients had mutations in *AXIN1*; 19 (2.7%) patients had mutations in *AXIN2* and 22 (3.1%) had mutations in the β-catenin gene. Regarding the PI3K pathway, the *PIK3CA* gene was mutated in 104 (14.7%) patients and 36 (5.1%) patients also had mutations in *PTEN*.

Taking into consideration that patients could have a mutation in both the Wnt and PI3K pathway genes simultaneously, we stratified patients and grouped them by: (1) having no mutations in any of the pathway genes; (2) mutated for the PI3K pathway genes and wild-type Wnt pathway genes; (3) having mutations in Wnt pathway genes and wild-type PI3K pathway genes, and (4) having mutations in the genes of both pathways simultaneously. We found that 236 (33.5%) patients had no mutations in any of the pathways. Fifty-six (7.9%) patients had mutations in PI3K pathway genes, and 346 (49.1%) patients had mutations in Wnt pathway genes, while 67 (9.5%) patients had mutations in the genes of both pathways. Then, we verified whether these mutations could influence patient survival by using Kaplan Meier curves (Supplementary Material 1). There were no significant differences in overall survival between the subgroups of patients and the mutations analyzed.

### Association of PI3K and Wnt/β-catenin genetic alterations with clinicopathological characteristics in CRC patients

Next, we analyzed the correlation between the frequency of genetic alterations in these pathways and a subset of clinicopathological parameters. In a comparison of patients without mutations and those with mutations in PI3K alone, PI3K mutations were more frequently associated with early stages (I and II) of CRC progression (56.4% vs. 32.1%; p = .00619). Regarding those with alterations in the Wnt pathway alone, we observed that these patients were more likely diagnosed at ages above 50 years (94.7% vs. 89.4%; p = .045); and in the early stages (I and II) (43.4% vs. 32.1%; p = .015). There was also a positive association with tumor location in the rectum (34.1% vs. 18.9%; p = .00022). Furthermore, we detected a higher frequency of patients with mutations in Wnt pathway genes associated with microsatellite stability (MSS) (94.3% vs. 83.7%; p = .00507). We did not observe significant clinical differences between patients with mutations in the genes of both pathways and patients with no mutations in these genes (Table 1). These results suggest that genetic alterations in these pathways may be associated with early clinicopathological characteristics in CRC patients.

**Table 1.** Clinicopathological characteristics of patients correlated with the pathway mutational status.

		None	PI3K	Chi-squared p value	WNT	Chi-squared p value	Both (PI3K and WNT)	Chi-squared p value
Number of patients		236 (33,5%)	56 (7,9%)		346 (49,1%)		67 (9,5%)	
Sex				0.939		0.614		0.838
	Female	113 (49,1%)	21 (51,2%)		147 (51,8%)		31 (51,7%)	
	Male	117 (50,9%)	20 (48,8%)		137 (48,2%)		29 (48,3%)	
Tumor Location				0.128		0.00022 *		0.765
	Right	103 (50%)	27 (67,5%)		102 (34,8%)		30 (46,9%)	
	Left	64 (31,1%)	8 (20%)		91 (31,1%)		23 (35,9%)	
	Rectum	39 (18,9%)	5 (12,5%)		100 (34,1%)		11 (17,2%)	
Stage				0.00619 *		0.015 *		0.196
	I & II	72 (32,1%)	22 (56,4%)		111 (43,4%)		23 (42,6%)	
	III & IV	152 (67,9%)	17 (43,6%)		145 (56,6%)		31 (57,4%)	
Age of Onset				0.42		0.045 *		0.062
	Early Onset (<50)	24 (10,6%)	2 (5%)		14 (5,3%)		1 (1,7%)	
	Late Onset (>50)	203 (89,4%)	38 (95%)		250 (94,7%)		57 (98,3%)	
MSI				0.094		0.00507 *		0.606
	MSI	17 (16,3%)	14 (29,8%)		11 (5,7%)		11 (21,2%)	
	MSS	87 (83,7%)	33 (70,2%)		183 (94,3%)		41 (78,8%)	

Note: Significance was considered when * p < 0.05

To address whether these mutations could be prognostic factors of clinicopathological characteristics, we performed a multivariate regression analysis. Patients diagnosed with stage III and IV (OR: 0.38; 95%CI: 0.16–0.72; *p* = .05 for PI3K; OR: 0.60; 95%CI: 0.40–0.90; *p* = .015 for Wnt) were associated with lower odds ratios of mutations in the Wnt or PI3K pathway genes. On the other hand, patients with tumors in the rectum (OR: 2.64; 95%CI: 1.64–4.31; *p < *.001) had greater odds ratios of mutations in Wnt pathway genes. Other patient characteristics were not statistically significant (Table 2). This result suggests that among these clinicopathological parameters, mutations in these pathways could function as predictive factors of CRC.

**Table 2.** Multivariate regression analysis for prognostic assessment according to clinicopathological parameters.

	Odds Ratio	p value	95% Confidence Intervals	
**PI3K**				
Intercept	0.147	0.075	0.008	0.829
Age of Onset - Late	3.184	0.271	0.609	58.641
Stage III & IV	0.347	0.005 *	0.164	0.719
Tumor Location - Left	0.554	0.185	0.219	1.281
Tumor Location - Rectum	0.372	0.128	0.084	1.17
**WNT**				
Intercept	0.621	0.241	0.276	1.363
Age of Onset - Late	1.936	0.071	0.955	4.053
Stage III & IV	0.605	0.015 *	0.402	0.905
Tumor Location - Left	1.338	0.21	0.849	2.115
Tumor Location - Rectum	2.639	0.0000826 *	1.637	4.309
**Both (PI3K and WNT)**				
Intercept	0.052	0.006 *	0.003	0.289
Age of Onset - Late	6.511	0.071	1.3	118.479
Stage III & IV	0.607	0.12	0.323	1.147
Tumor Location - Left	1.353	0.398	0.665	2.725
Tumor Location - Rectum	1.329	0.497	0.569	2.976

Note: Significance was considered when * p < 0.05

### Activity analysis of the PI3K/Akt and Wnt/β-catenin pathways

To evaluate pathway activity in our study model, we initially verified the activation of the PI3K/Akt and Wnt/β-catenin pathways through treatment with IGF-1 and with conditioned medium of cells that overexpress Wnt3a (L-Wnt3a) or recombinant Wnt3a (Wnt3a-RH). W*e* observed that in HT-29 cells, IGF-1 treatment significantly increased p-Akt in the treatment (at 15, 30 and 60 min) and this effect persisted for 24–48 h. Since GSK3β is a downstream target of Akt,^18^ we then monitored p-GSK3β levels and observed a similar increase in phosphorylation from 15 min to 48 h. In HCT-116 cells, we observed a significant increase in Akt phosphorylation from 15 min to 48 h and an increase in GSK3β phosphorylation restricted to the 60 min time point. (Supplementary Material 2).

**Figure 1.**
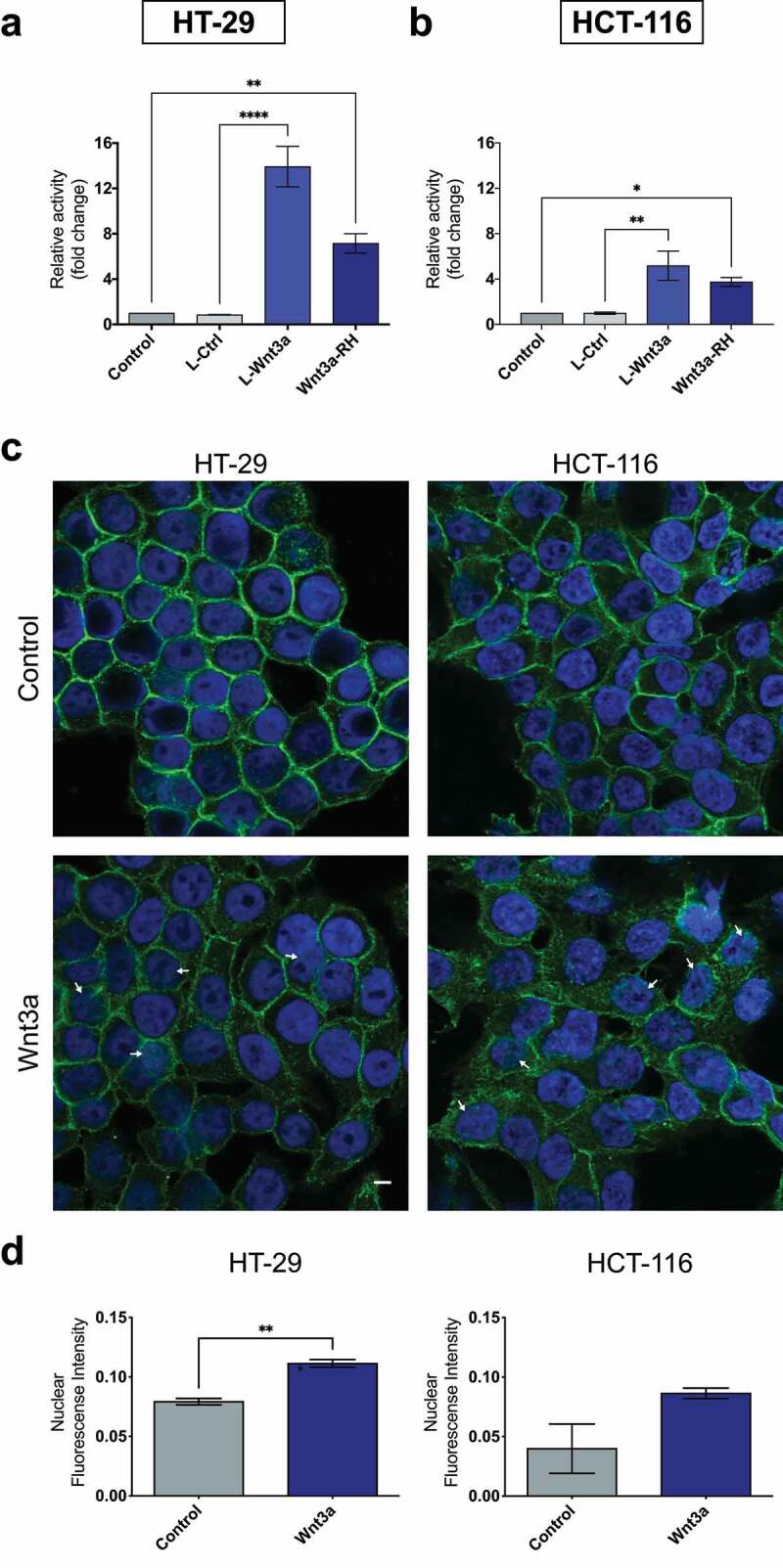
Treatment with Wnt3a leads to β-catenin nuclear translocation and transcriptional activity. Quantification of the transcriptional activity of β-catenin in CRC cell lines HT-29 (a) and HCT-116 (b). Cells were treated as indicated and for 24 h and the transcriptional activity was normalized to renilla quantification. Data are presented as the mean ± SEM of three independent experiments. Significance was determined using ANOVA followed by Bonferroni’s post-hoc test (*p < .05, **p < .01, and ***p < .001). Wnt3a affects the subcellular localization of β-catenin in CRC cells (c and d). Immunofluorescence analysis of β-catenin (green) localization. Nuclei were stained using DAPI (blue). White arrows indicate β-catenin spots in the nuclei. The fluorescence intensity was quantified using ICY Bioimage Analysis software (Institut Pasteur, Paris, France). Data are presented as the mean ± SEM of three independent experiments. Significance was determined using ANOVA followed by Bonferroni’s post-hoc test (**p < .01). Scalebar equivalent to 20 μm.

Since most of CRC cell lines harbor mutations in different genetic components of the Wnt/β-catenin pathway that constitutively activate the pathway, we evaluated whether these cells were responsive to treatment using Wnt3a, a ligand of this signaling pathway. We observed that cells treated with recombinant Wnt3a or with conditioned media rich in Wnt3a increased transcriptional activity of β-catenin in both cell lines (Figure 1a,b). We further confirmed this result by monitoring the nuclear localization of β-catenin in both cells after the treatments and observed that Wnt3a stimulated significant translocation of the protein to the nucleus in the HT-29 cells and a trend in HCT-116; however, the protein was also observed in the cytoplasm. On the other hand, the control group showed β-catenin mainly at cell-cell contacts (Figure 1c,d).

Considering the small variations observed in the activation status of the PI3K/Akt and Wnt/β-catenin pathways in response to the treatments in HT-29 and HCT-116 cells, we expanded our evaluation to three more CRC cell lines (Caco-2, Lovo and SW480). Treatment with Wnt3a, IGF1, or both combined resulted in a wide range of activation of these pathways (Supplementary Material 3 and 4), indicating that this cross activation happens in a cell-specific manner. Therefore, we choose HT-29 and HCT-116 cells to continue our analysis.

### Treatment with Wnt3a and IGF1 activates the PI3K/Akt pathway

**Figure 2.**
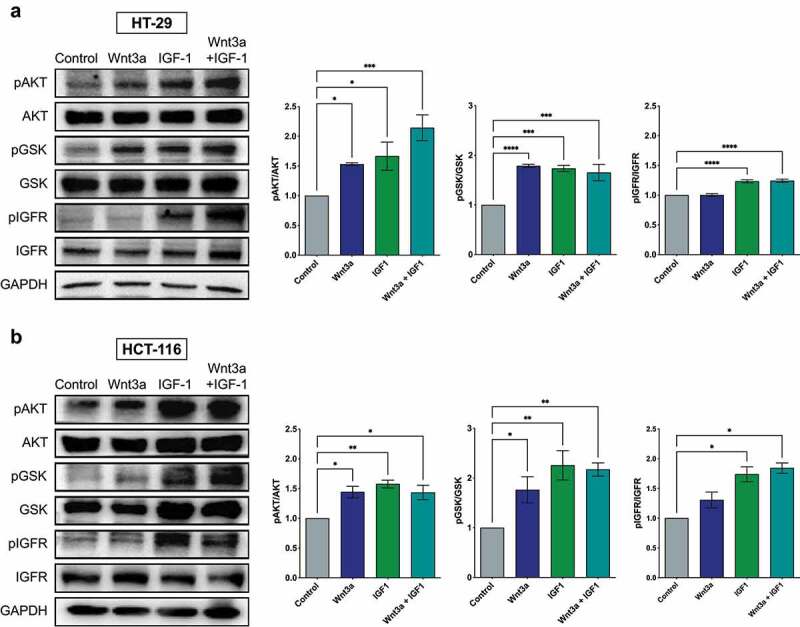
Activation of the PI3K/Akt pathway after treatment with Wnt3a, IGF1, or Wnt3a and IGF1 in CRC cells. Western blotting of pAkt, total Akt, pGSK, total GSK3β, and total IGFR in CRC cell lines HT-29 (a) and HCT-116 (b). The graphs are presented as a densitometric analysis of the ratio between the phosphorylated protein and its total protein expression. Data are presented as the mean ± SEM of three independent experiments. Significance was determined using ANOVA followed by Bonferroni’s post-hoc test (*p < .05, **p < .01 and ***p < .001).

Some studies have shown that when the PI3K/Akt and Wnt/β-catenin pathways are concomitantly activated, synergism in tumor progression is observed. To test this hypothesis in our study models, HT-29 and HCT-116 cells were treated with Wnt3a or IGF1, or simultaneously with both treatments, and the activation of these pathways was analyzed through the phosphorylation of key proteins. Our data show that Wnt3a and IGF1 individually or in combination, all stimulated the phosphorylation of Akt and GSK3β in the cell lines indicating increased activity of these pathways. It is worth mentioning that the activation of the IGFR receptor was noticed only with the IGF1 treatment alone and in the combination with Wnt3a, indicating that Wnt3a acts by increasing the phosphorylation of Akt through a different receptor (Figure 2a,b).

### Treatments with Wnt3a and IGF1 activate the Wnt/β-catenin pathway and combined treatment with these agents potentiates this effect

**Figure 3.**
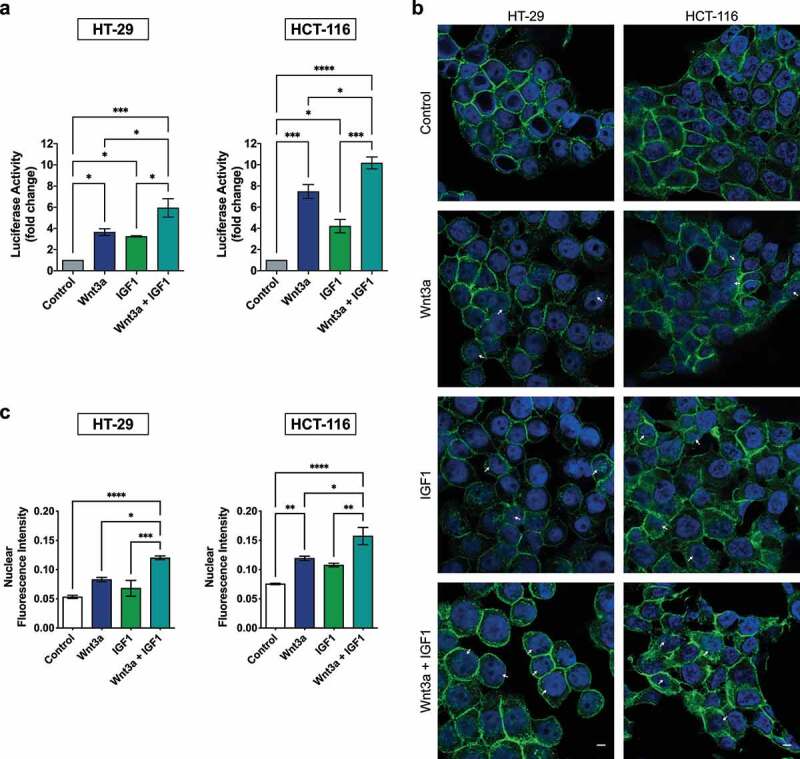
Treatment with Wnt3a or IGF1 activates the Wnt/β-catenin pathway and the combined treatment of these agents potentiates this effect. (a) Transcriptional activity of β-catenin after respective treatments. Data are presented as the mean ± SEM of three independent experiments. Significance was determined using ANOVA followed by Bonferroni’s post-hoc test (*p < .05, **p < .01 and ***p < .001). (b) Immunofluorescence of β-catenin (green) in CRC cells after the indicated treatments. The nucleus was stained using DAPI (blue). White arrows indicate β-catenin spots in the nuclei. (c) The fluorescence intensity quantification was analyzed using ICY Bioimage Analysis software. Data are presented as the mean ± SEM of three independent experiments. Significance was determined using ANOVA followed by Bonferroni’s post-hoc test (*p < .05). Scalebar equivalent to 20 μm.

The effect of the combination of treatment on the Wnt/β-catenin pathway was then analyzed. As expected, Wnt3a alone was able to activate the pathway in both HCT-116 and HT-29 cells. An increase in the transcriptional activity of β-catenin was also noted after individual treatment with IGF1 when compared to the control. The combined treatment exhibited greater luciferase activity when compared to each individual treatment (Figure 3a). Additionally, immunofluorescence analysis corroborated these results, as Wnt3a treatment induced a significant nuclear translocation of β-catenin from the cell junctions. Treatment with both agents led to greater nuclear β-catenin fluorescence in both cells (Figure 3b,c).

Together, these results show that IGF1 can activate the PI3K/Akt pathway and, to some extent, the Wnt/β-catenin pathway. Likewise, Wnt3a was able to activate both pathways, indicating a crosstalk between them. Furthermore, the combination of treatment with these ligands potentiated the activation of these pathways.

### Simultaneous activation of the Wnt/β-catenin and PI3K/Akt pathways increases proliferation and cell migration

**Figure 4.**
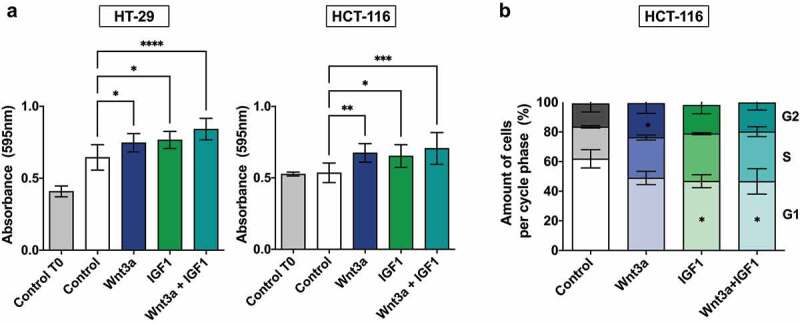
Cellular proliferation and cell cycle analysis after Wnt3a and IGF treatment. (a) Analysis of cellular proliferation of CRC cell lines by crystal violet assay. T0 represents cells without treatment at the 0 h time point. Data are presented as the mean ± SEM of three independent experiments. Significance was determined using ANOVA followed by Bonferroni’s post-hoc test (*p < .05, **p < .01 and ***p < .001). (b) Quantitative determination of cell cycle phases in HCT-116 cells. Data are presented as the mean of two independent experiments. Significance was determined using ANOVA followed by Bonferroni’s post-hoc test (*p < .05).

To determine if the different treatments affected the malignant phenotype, we evaluated the effect of the treatments on the proliferation and migration of HT-29 and HCT-116 cells. Individual activation of the pathways using Wnt3a and IGF1 or combined treatment with these agents led to an increase in cell proliferation. (Figure 4a). We verified, through flow cytometry in HCT-116 cells only, that IGF1 alone and the combined treatment induced a significative reduction of cells in the G1 phase (p = 0,0294) and an increase of cells in G2 phases for the Wnt3a treatment (p = 0,0007) (Figure 4b).

**Figure 5.**
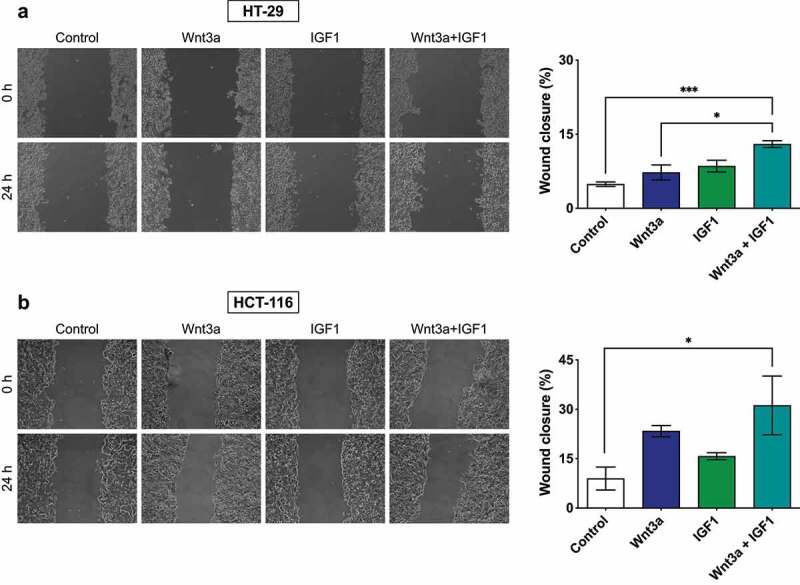
Migratory analysis of HCT-116 and HT-29 CRC cells. Cells were grown until confluence and subjected to a wound-healing assay. Bar graphs represent percentages of cell migration. Data are presented as the mean ± SEM of three independent experiments. Significance was determined using ANOVA followed by Bonferroni’s post-hoc test (*p < .05, **p < .01 and ***p < .001).

Next, we analyzed cell migration and observed that the combined treatment with both compounds significantly stimulated the migratory potential of both cell lines. In the HT29 cell line the combined treatment also showed a significant increase when compared to the Wnt3a treatment (Figure 5). Together, these data suggest the involvement of the PI3K/Akt and Wnt/β-catenin pathways in cellular proliferation, and a potentiating effect of both pathways toward a more migratory phenotype.

### Pharmacological inhibition of the PI3K/Akt pathway using LY294002

**Figure 6.**
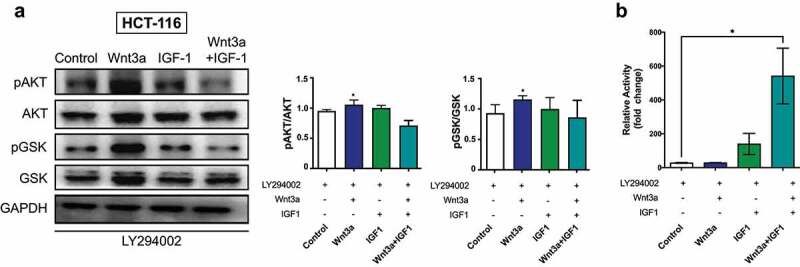
Pharmacological inhibition of PI3K/Akt pathway using LY294002. (a) Western Blotting of pAkt, total Akt, pGSK3β and total GSK3β in HCT-116 cells after respective treatments. (b) The graphs are presented as a densitometric analysis of the ratio between the phosphorylated protein and its total protein expression. (c) Transcriptional activity of β-catenin using a Luciferase assay. Data are presented as the mean ± SEM of three independent experiments. Significance was determined using ANOVA followed by Bonferroni’s post-hoc test (*p < .05, **p < .01 and ***p < .001).

Our next step was to assess the impact of PI3K/Akt pathway inhibition, using the specific inhibitor LY294002 (LY), on the effects of the treatments previously described. For this assay, HCT116 cells were chosen because of their high sensitivity to the ligands. We observed that LY prevented the increase in phosphorylation of Akt and GSK3β induced by IGF1 and by the combined treatment using IGF1 and Wnt3a. However, LY did not affect the Akt phosphorylation induced by Wnt3a, indicating that this ligand phosphorylates Akt and GSK3β through a PI3K-independent mechanism (Figure 6a). Regarding β-catenin transcriptional activity, PI3K inhibition using LY prevented the previously described increases after treatment with Wnt3a or IGF1 (see Figure 2a). Surprisingly, LY induced a significantly stronger transcriptional activity of β-catenin when cells were treated with Wnt3a and IGF1 (Figure 6b).

**Figure 7.**
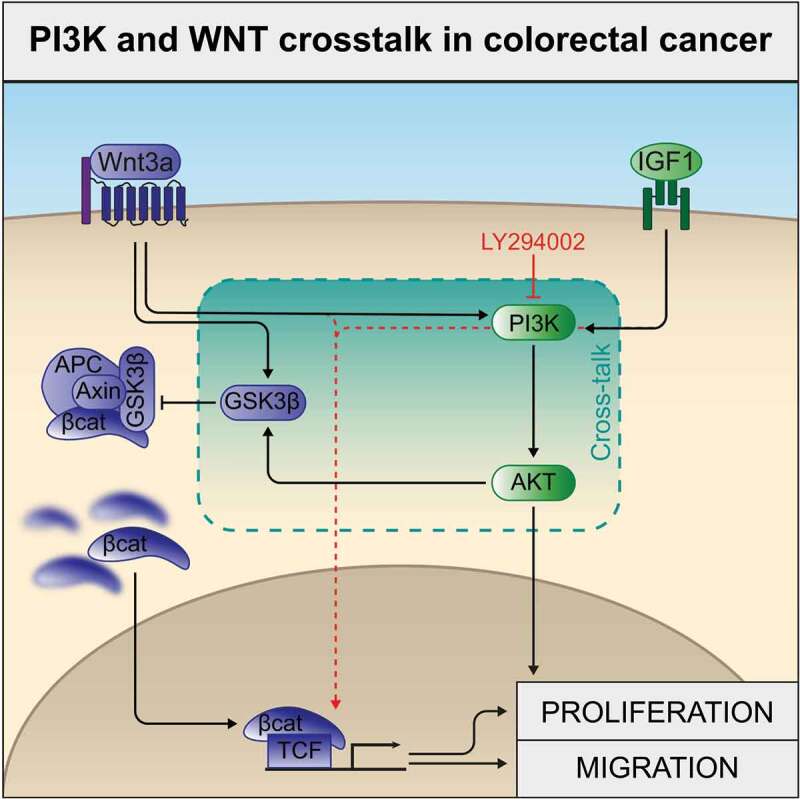
Schematic diagram of PI3K and WNT crosstalk in colorectal cancer. Wnt3a activating its receptor leads to GSK3β phosphorylation, hindering the formation of the destructive complex, and promoting increased β-catenin availability in the cytoplasm (blue pathway). IGF1 interacts with its receptor with subsequent PI3K and AKT activation (green pathway). In the article we show that both pathways promote cellular proliferation and migration, and crosstalk through PI3K activation (exemplified with LY294002 inhibition results/red arrows indicate activation of alternate pathways upon inhibition of PI3K in the combined treatment).

In Figure 7 we summarize our findings regarding the crosstalk of Wnt3a and IGF1 pathways. Wnt3a after interacting with its receptor leads to the phosphorylation of GSK3β and activation of PI3K. GSK3β phosphorylation hinders the formation of the destructive complex and provides greater availability of β-catenin in the cytoplasm. After being translocated to the nucleus, β-catenin binds to TCF and promotes transcription of genes which will act over proliferation and migration of these cells (pathway in blue). Wnt3a treatment also led to the activation of the PI3K/Akt, a similar effect when compared with the treatment with IGF1 alone (green pathway). Pharmacological inhibition of PI3K (shown in red) hindered the proliferation and migration of the cells upon single treatment (IGF1 or Wnt3a) showing that these effects are up to a certain point PI3K-dependent. However, PI3K inhibition associated with the combined treatment (Wnt3a plus IGF1) resulted in the highest transcriptional activity of β-catenin. This suggests that these pathways may redirect their signaling to additional pathways (red arrows) in order to enhance β-catenin activity.

## Discussion

The prognostic role of genetic alterations of PI3K/Akt or Wnt/β-catenin pathways in order to search for therapeutic candidates that complement activity studies has been few explored in CRC. In the present study, we initially analyzed the mutational status of both pathways and their prognostic implications in a large set of CRC patient data from publicly available databases. Our results showed no differences in overall survival among the groups. However, the accumulation of other mutations, which was not examined in this study, might influence these results. For instance, the *TP53* gene is frequently dysregulated in CRC and is associated with resistance to current therapies leading to poor prognoses.^19^ Tumors with *KRAS* and *BRAF* mutations are also associated with worse prognoses in CRC patients. In addition, a large meta-analysis including over 12,000 patients showed that *PIK3CA* mutations were not significantly associated with CRC.^5^ On the other hand, mutations in *APC* seem to have a central impact on the survival of CRC patients.^20^ Furthermore, our data showed an association between mutations in the PI3K/Akt or Wnt/β-catenin pathway genes and colorectal cancer age of onset and early stages, suggesting that mutations in the genes of these pathways might be useful as prognostic predictive markers and/or as therapeutic targets to improve outcomes in CRC treatment. Studies in lung cancer indicate that the activation of these pathways may contribute to development of new primaries and chemoresistance in relapsed small-cell lung cancer.^21,22^ It is important to acknowledge that these alterations are result of a broad mutational analysis grouping mutations that might have diverging effects in tumoral progression. A larger cohort of patients would enable a more detailed analysis of specific mutations and combinations of such.

We also analyzed the activity of the PI3K/Akt and Wnt/β-catenin pathways using CRC cell lines, HT-29 and HCT-116, treated with IGF1, Wnt3a, or both combined. We observed that IGF1 increased the phosphorylation of Akt and GSK3β in relation to non-treated cells, suggesting that cells with mutations in *PI3KCA* continue to be activated in the presence of its ligand. The same effect was observed in other CRC cell lines^23^ where PTEN plays a dominant role. PTEN reverts PIP3 back to PIP2, shifting *PI3KCA* activating mutations to an Akt-independent mechanism.^24^ In addition, cells treated with Wnt3a increased the nuclear β-catenin and its transcriptional activity, indicating that it remains ligand-dependent. Some studies suggest that PI3K/Akt is not involved in the Wnt/β-catenin pathway,^25,26^ however our results show otherwise, suggesting crosstalk between these pathways. Corroborating these results, cells overexpressing Wnt have been reported to cause an increase in the phosphorylation of Akt.^27,28^ Furthermore, simultaneous treatment with IGF1 and Wnt3a induced a potentiating effect on the activation of these pathways. Indeed, concomitant activation of both pathways potentiates tumor progression in ovarian tumor^16^ as well as CRC,^17^ which reinforces our hypothesis that interplay between these pathways contributes to CRC progression. The fact that IGF1 and Wnt3a induced a variable response in other CRC cell lines, as shown here, indicates that mutations in *PIK3CA, APC*, and *CTNNB1*, as is the case in HT-29 and HCT-116 cells,^29,30^ are necessary for the ligand-mediated potentiating effect to occur.

We observed an increase in cell proliferation after treatment with these ligands, but this effect was cell-specific, given that Wnt3a was more effective in HCT-116 cells and IGF1 was more effective in HT-29 cells. This differential effect can be explained by the abundance of specific receptors of these ligands.^31^ The increase in cell proliferation was confirmed by cell cycle analysis, where we observed a major portion of cells in the S and G2 phases after treatment. Also, we showed that when both pathways were simultaneously activated, cells migrated more when compared to the control group. Indeed, PI3K regulates cell migration by direct binding of the protein to its lipid products or indirectly via crosstalk with Rho GTPases.^32^ On the other hand, the Wnt/β-catenin pathway contributes to cell migration through E-cadherin redistribution, cytoskeleton rearrangement, and an increase in Rho family proteins.^33^ Additionally, synergism among mutations in the genes of both pathways during the development of intestinal cancers has previously been described promoting tumor aggressiveness in preclinical studies.^34^

In order to clarify the crosstalk and potentiating effects observed in this study, we used LY294002, a specific inhibitor of PI3K^35^ and found that it inhibited the transcriptional activity and nuclear localization of β-catenin. The increased transcriptional activity of β-catenin obtained by the association of LY, Wnt3a, and IGF1 might have occurred due to a shift in the activation of IGF1 toward the MAPK pathway, which, in combination with Wnt3a, may induce increased activity. The interaction between Wnt/β-catenin and MAPK pathways has already been described.^36^ However, further studies are necessary to confirm this hypothesis. In relation to Wnt/β-catenin, it is possible that Wnt3a stimulates the phosphorylation of Akt and GSK3β by an independent mechanism of PI3K. A previous study showed that Wnt3a activates ROCK and directly phosphorylates GSK3β in a dependent form of the non-canonical Wnt pathway.^37^ Furthermore, our results showed that the PI3K inhibitor prevented the increase in proliferation and migration induced by the individual or combined treatment using Wnt3a and IGF, suggesting that these pathways cooperate during tumor progression. A synergic effect between the Wnt/β-catenin and PI3K/Akt pathways during development and progression has previously been observed in ovarian cancer and CRC.^16,34^

Several therapies have already been developed that target components of the Wnt/β-catenin or PI3K pathways. Despite showing good results in clinical trials, resistance to these therapies is frequently observed.^38^ The crosstalk between pathways allows for signaling redirection and ultimately the emergence of resistance. An example of this is the fact that patients with mutations exclusively in one of these pathways are found predominantly in the early stages of tumor progression. Our results reinforce the need for mutational status assessment of WNT and PI3K pathways in clinical biopsies and for those patients with concomitant mutations, the usage of clinical approaches targeting proteins located downstream of PI3K.

In conclusion, we have provided evidence for the value of targeting key proteins of the PI3K/Akt and Wnt/β-catenin pathways and their prognostic implications for developing new directed therapies and improve outcomes in CRC treatment. In addition, our findings highlight the existence of inter-connectivity between these two crucial signaling pathways in CRC progression (Figure 7), and that this interplay seems to be largely PI3K-dependent. Future studies are indispensable to establish the translational impact of this interrelationship mechanism between these two pathways in this cancer type.

## Materials and methods

### Materials

Rabbit anti-pAkt S473, anti-Akt, anti-pGSK3Β S9, anti-GSK, anti-IGF1R and anti-pIGF1R Y1135 monoclonal antibodies were purchased from Cell Signaling (Danvers, MA, USA). Mouse anti-GAPDH monoclonal antibody was purchased from Ambion (Carlsbad, CA, USA). Alexa Fluor® 488 goat anti-mouse IgG was obtained from Molecular Probes (Eugene, Oregon, USA). Horseradish peroxidase (HRP)-conjugated anti-mouse and anti-rabbit IgG were acquired from Sigma (Saint Louis, MO, USA). Mouse anti-β-catenin was purchased from Thermo Fisher (Waltham, MA, USA). Insulin-like growth factor 1 (IGF-1) was obtained from Cell Signaling and reconstituted in 20 mM citrate, pH 3.0, to obtain a stock concentration of 100 µg/mL. The 2-(4-morpholinyl)-8-phenyl-1(4 H)-benzopyran-4-one LY294002 (PI3K inhibitor) was obtained from Cell Signaling and diluted in dimethyl sulfoxide (DMSO) to obtain a stock concentration of 3 mg/mL. Recombinant Wnt3a was purchased from R&D Systems (Minneapolis, MN, USA) and diluted in phosphate-buffered saline (PBS) + 0,1% bovine serum albumin (BSA) to obtain a stock concentration of 200 µg/mL and a work concentration of 50 ng/mL.

### Cell culture and treatments

The human CRC cell lines HT-29 (ATCC, HTB-38), HCT-116 (ATCC, CCL-247), Caco-2 (ATCC, HBT-37), SW480 (ATCC, CCL-228), LoVo (ATCC, CCL-229), L-Wnt3a (ATCC, CRL-2647) and the parental line L-cells (ATCC, CRL-2648) were obtained from American Type Culture Collection (Manassas, VA, USA). The cells were maintained in Dulbecco’s modified Eagle’s medium (DMEM – Invitrogen Corporation, Carlsbad, CA, USA). SW480 cell line was grown in DMEM/ Ham′s Nutrient Mixture *F12* and LoVo cell line was maintained in Roswell Park Memorial Institute (RPMI) 1640 medium. All mediums were supplemented with 10% fetal bovine serum (FBS), penicillin G (100 mg/L), and streptomycin (60 mg/L) (Invitrogen) and cultivated at 37°C in a humidified atmosphere containing 5% CO_2_. Cells were passaged weekly by using a solution of 0.05% trypsin/0.02% EDTA in PBS. A table with the most noticeable mutations already described for each cell line is provided as Supplementary Material 5.

Prior to pharmacological treatments, cells were maintained overnight in DMEM with 1% FBS and then treated with Wnt3a (100 ng/mL), 50% conditioned medium of Wnt3a, or IGF1 (100 ng/mL) for different times. For experiments with the PI3K inhibitor, cells were pre-treated with 15 µg/mL of LY294002 1 h before the treatments described above.

### Conditioned medium Wnt3a production

Conditioned medium (CM) of Wnt3a was produced from L-Wnt3a and the parental L-cells cell lines, both kindly provided from Dr Jose Garcia Abreu from Universidade Federal do Rio de Janeiro. These cells were cultured in DMEM medium, and the CM was collected as previously described.^39^ Briefly, cells at 80% confluence were sub-cultured 1:10 in T75 bottles with 10 mL of DMEM medium and grown for 4 days. The medium was collected, centrifuged for 1000 g for 10 min and filtered (0.22 µm pore). Another 10 ml of medium was added, and the cells were cultured for further 3 days. The medium was collected again, centrifuged for 1000 g for 10 min and filtered (0.22 µm pore). The first and second batch were mixed 1:1 and frozen for posterior use.

### Protein lysates obtention and Western blotting

To obtain total protein lysates, the cells were homogenized in RIPA extraction buffer: 1% Triton X-100; 0.5% sodium deoxycholate; 0.2% SDS; 150 mM sodium chloride, pH 7.4; 20 mM sodium fluoride; containing 1 mM sodium orthovanadate; and a cocktail of protease inhibitors (Sigma) for 30 min at 4°C. The extract was centrifuged at 10000 g and 4°C for 10 min. The supernatant was collected and stored at −80°C. The protein extract was dosed using the commercial BCA kit (Bioagency Biotecnologia Ltda, São Paulo, Brazil) and the BSA protein (Sigma) was used as standard. Protein lysates were submitted to electrophoresis in a polyacrylamide gel containing sodium dodecyl sulfate (SDS-PAGE) and transferred to nitrocellulose membranes using the Trans-Blot Semi-Dry Transfer Cell equipment (Bio-Rad Laboratories, Hercules, CA, USA). Subsequently, the membranes were blocked with 5% BSA diluted in 0.1% TBS-Tween for 1 h and incubated overnight with the following primary antibodies: anti-GSK3β (1: 1000), anti-pGSK3β S9 (1:1000), anti-Akt (1:1000), anti-pAkt S473 (1:1000), anti-IGF1R (1:1000) or anti-pIGF1R Y1135 (1:1000). Anti-GAPDH (1:50.000) was incubated for 1 hour. After successive washes, the membranes were incubated for 1 h with corresponding secondary antibodies, anti-mouse or anti-rabbit conjugated to peroxidase, at a dilution of 1:40000 in 0.1% TBS-T, for 60 min. The membranes were washed with 0.1% TBS-T and the signal was detected by chemiluminescence using the ECL kit (Amersham Biosciences GE Healthcare, Buckinghamshire, UK).

### TCF/LEF transcriptional-activity reporter assay

To analyze the transcriptional activity of β-catenin, we measured the TCF/LEF transcription-factor reporter activity by using a Dual-Luciferase Reporter Assay System Kit (Promega, Madison, WI, USA). Two TCF luciferase reporters were used in the assay: an intact wild-type TCF-luciferase reporter construct (Super 8X TOPflash), and a mutated TCF-luciferase reporter construct (Super 8X FOPflash), used as a negative control. The cells were seeded in 24-well plates, grown until subconfluence and then were co-transfected transiently with 2 μg each of Super 8X TOPflash β-Catenin Luciferase reporter and pRL-TK (Renilla luciferase) plasmids or Super 8X FOPflash β-Catenin Luciferase reporter and pRL-TK plasmids. Transfections were performed using FuGENE® HD Transfection Reagent (Roche, Pleasanton, CA). After 24 h of transfection, cells were washed twice with PBS, FBS-depleted for 24 h, and then subjected to treatments. After treatments, extracts were prepared using 200 μL of reporter lysis buffer (Promega). Renilla and firefly luciferase activities were assayed using the Dual-Luciferase Reporter Assay System Kit (Promega), according to manufacturer instructions. The Veritas plate luminometer (Tuner Biosystems, Sunnyvale, CA, USA) was used to perform the reading. The firefly luciferase activity in each well was normalized relative to the Renilla luciferase activity.

### Immunofluorescence

Cells were cultured on glass coverslips in a 12-well plate, and after treatments, rinsed twice with PBS and fixed with 4% paraformaldehyde in PBS/CM (PBS containing 100 mM CaCl2 and 100 mM MgCl2, pH 8.0) for 10 min at room temperature. The coverslips were washed in PBS/CM, permeabilized with 0.2% BSA + 0.1% Triton X-100 in PBS-CM for 30 min at room temperature and then blocked with 0.2% BSA in PBS for 60 min. Coverslips were once again washed with PBS and then incubated with primary β-catenin antibody diluted 1:500 in 0.2% BSA in PBS, overnight at 4°C. Subsequently, the cells were washed, and the secondary antibody anti-mouse conjugated to Alexa fluor 488 (1:200) was added for 1 h. The coverslips were rinsed and incubated with DAPI (1:1000) for 1 min, then the slides were mounted in Antifade ProLong Gold reagent (Invitrogen). All images were captured using a confocal laser microscope (Fluoview FV10i Overview, Olympus, USA) and analyzed with the ICY bioimage analysis software. To quantify nuclear β-catenin, we examined merged fluorescence images for β-catenin co-localization with DAPI labeling; at least 100 cells were analyzed per condition, and the nuclear β-catenin amount was quantified using ImageJ software.

### Cell proliferation

Cell proliferation was measured using the crystal violet method. The cells were seeded in a 96-well plate at a final concentration of 2x10^4^/well. The next day, the medium was replaced, and the cells were maintained overnight in DMEM with 1% FBS. Then, cells were treated with Wnt3a or IGF1 in DMEM with 1% FBS for 24 h. After this time, cells were washed with PBS (pH 7.0), fixed with ethanol for 10 min, stained with crystal violet solution (0.05% crystal violet and 20% methanol) for 10 min, washed twice with water and then, the remaining crystal violet was solubilized with methanol for 5 min. Absorbance was measured at 595 nm with a Spectra Max 190 spectrophotometer (Molecular Devices, Sunnyvale, CA, USA).

### Flow cytometry

For cell cycle analysis, cells (2 x 10^4^) were cultured in T25 flasks, FBS-depleted overnight and treated with Wnt3a and/or IGF1 for 24 h. Cells were then harvested through trypsinization, washed once with PBS, and then kept in 70% ethanol for 30 min at 4°C. The cells were washed with ice-cold PBS and stained with 75 μM propidium iodide (PI) for 10 min in a buffer containing 3.4 mM Tris–HCl (pH 7.6), 10 mM NaCl, 0.2% Triton X-100, and 3500 U/L RNase. DNA content was examined by collecting 10000 events for cell cycle analyses by using a FACSCalibur flow cytometer (BD Transduction Labs, Lexington, KY) and Mod Fit LT software.

### Cell migration assay

The cells were plated into a 12-well plate and allowed to grow until they reached confluence. Mitomycin C (10 μg/μL) was added for 1 h and then monolayers were scraped by a sterile pipette tip to perform the wound healing assay. For each well, five sites of a unique regular wound were analyzed under an Axio Observer Z1 microscope (Carl Zeiss, Inc., Jena, Germany), selected and marked. Then, the cells were permitted to migrate into the clearing area for 24 h. Cells were photographed immediately after wounding (0 h) and at the end of the experiment (24 h). The lesion area was manually quantified using ImageJ software.

### CRC data

The CRC data was acquired from the cbioportal.org website,^40^ which is an open-access resource for cancer genomics datasets. The dataset was queried by genes of interest based on the most common mutations in the Wnt pathway (*APC, CTNNB1, AXIN2* and *AXIN1*) and in the PI3K pathway (*PI3KCA, PTEN, PIK3R1* and PIK3R2). The studies DFCI;^41^ Genentech;^42^ TCGA Firehose Legacy;^43^ MSK;^44^ CaseCCC;^45^ and CPTAC-2;^46^ were included with a total of 705 patients in the study. Cases classified as high microsatellite instability (MSI) were removed, as patients with this phenotype have better survival than those with MSS, which could influence the results.^47^ Metastatic samples were also excluded. Only driver mutations were considered because of their already described biological effects. The study was adjusted by the Cox model, stratified by wild-type or mutated Wnt, wild-type or mutated PI3K and simultaneous Wnt + PI3k pathway mutations. An overview of clinicopathological data might be observed in Table 2.

### Statistical analysis

Quantitative data are expressed as means ± SEM from at least three independent experiments. Statistical analyses were performed, and column graphs were created using GraphPad Prism 9.0 software (GraphPad Software, San Diego, CA, USA). The normality test (Shapiro–Wilk test and D’agostin-pearson) was used to determine whether sample data had normal distribution using R Project for Statistical Computing software version 3.6.2 (The R Foundation). For parametric analysis, one-way variance analysis (ANOVA) followed by the Bonferroni posttest was used for comparisons between treatments. For non-parametric analysis, the Kruskal–Wallis test followed by Dunn’s test was used for comparisons between groups. Differences were considered significant when *p < .05; **p < .01; and ***p < .001.

For the CRC data, the statistical tests were performed using R Statistical Software (R Foundation for Statistical Computing, Vienna, Austria). For descriptive statistics, the absolute number with proportion was used. The chi-square test was applied for comparisons among the groups. The multivariate logistic regression model was done including the following covariates: onset, stage, and location. For the survival analysis, first, known microsatellite instability (MSI) patients were excluded as they show better prognosis compared to patients with microsatellite stable (MSS) tumors,^47^ then Kaplan Meier curves were performed for patients whose vital status and survival time data were available (644 patients) and grouped by the mutational status of the Wnt/β-catenin, PI3K/Akt or both pathways. Log-rank test was used to calculated differences among groups.

## Supplementary Material

Supplemental Material

## Data Availability

The data that support the findings of this study are available from the corresponding author upon reasonable request.
